# Aggressive clinical approach to obesity improves metabolic and clinical outcomes and can prevent bariatric surgery: a single center experience

**DOI:** 10.1186/s40608-017-0147-3

**Published:** 2017-02-21

**Authors:** Flavio A. Cadegiani, Gustavo C. Diniz, Gabriella Alves

**Affiliations:** 1 Division of Endocrinology and Metabolism, Department of Medicine, Escola Paulista de Medicina, Universidade Federal de São Paulo, R. Pedro de Toledo 781, 04039-032 São Paulo, SP Brazil; 2 Corpometria Institute, an Obesity and Endocrinology Center, SGAS 915 Centro Clínico Advance Salas 260/262/264, 70390-150 Brasilia, DF Brazil

**Keywords:** Obesity, Pharmacologic therapy, Behavioral strategies, Dietary adherence, Exercise intervention, Liraglutide, SGLT2, Bariatric surgery

## Abstract

**Background:**

The number of bariatric procedures has exponentially increased in the past decade, as a result of the lack of successful clinical weight-loss interventions. The main reasons for the failure of clinical obesity management are: (1) anti-obesity medications are administered as monotherapies (or pre-combined drugs); (2) lack of combination between pharmacotherapy and non-pharmacological modalities; (3) short duration of pharmacotherapy for obesity; (4) lack of weight-loss maintenance strategies; (5) misunderstanding of the complex pathophysiology of obesity; and (6) underprescription of anti-obesity medications. We developed a protocol that can potentially overcome the drawbacks that may lead to the failure of clinical therapy for obesity. The aim of this study is therefore to report the clinical and metabolic effects of our proposed obesity-management protocol over a 2-year period, and to determine whether this more intensive approach to obesity management is feasible and a possible alternative to bariatric surgery in patients with moderate-to-severe obesity.

**Methods:**

This retrospective study involved 43 patients in whom bariatric surgery was indicated. Patients underwent an intensive anti-obesity protocol that included pharmacotherapy with multiple drugs; intense surveillance with monthly body analysis by air-displacement plethysmography, electrical bioimpedance, and 3D body scans; weekly psychotherapy; diet planning with a dietician every 2 months; and exercises at least 3 times a week with exercises prescribed by a personal trainer at least once a month. Body weight (BW), total weight excess (TWE), obesity class, body mass index, fat weight, muscle weight, waist circumference, and visceral fat were analyzed. Markers of lipid and glucose metabolism, liver function, and inflammation were also evaluated. Therapeutic success was defined as >20% BW loss or >50% decrease in TWE after 1 year.

**Results:**

Significant improvements were observed in all clinical and metabolic parameters. Thirty-eight (88.4%) patients achieved 10% BW loss, and 32 (74.4%) achieved 20% BW loss. TWE decreased by >50% in 35 (81.4%) patients. Forty (93.0%) patients were able to avoid bariatric surgery.

**Conclusion:**

An intensive clinical approach to obesity management can be an effective alternative to bariatric surgery, although further randomized controlled studies are necessary to validate our findings.

## Background

In recent years, the number of bariatric procedures has increased, and almost 200,000 bariatric surgeries were performed in the USA in 2015 [1]. The prevalence of obesity has exponentially increased in the past decade, particularly, that of the severest classes of obesity for which current clinical approaches are not effective. Surgical intervention for moderate and severe obesity, when correctly recommended, leads to significant improvement in metabolic parameters [2, 3], remission of diabetes [2], improvement in beta-cell function [2] and normalization of glucose levels [3] as well as improvements in inflammatory markers [4], reduction of long-term cardiovascular risk [4, 5], cancer risk [6, 7], long-term mortality [5, 8], and moreover, it is a safe procedure [9]. These outcomes justify the formal recommendation of bariatric surgery for patients with a body mass index (BMI) of >40 kg/m^2^ (or >35 kg/m^2^ in the presence of comorbidities), according to the latest guidelines jointly issued by The Obesity Society (TOS), the American Heart Association (AHA), and the American College of Cardiology (ACC) [10].

The same guidelines also recommend that surgical intervention should be recommended only after the patient has: (a) attempted, unsuccessfully, to lose weight via clinical obesity-management strategies for at least 2 years, (b) been made aware of the lifelong limitations he/she will face after undergoing bariatric surgery, and (c) achieved a weight loss amounting to at least 5% of the total body weight (BW). However, the above requirements are not always followed by health-care providers [11, 12], and many individuals who undergo bariatric surgery have not previously attempted to lose weight clinically, have not been assessed by a multidisciplinary team [12, 13], and are not aware of the patient’s role, responsibilities, and limitations after the surgery [13, 14].

The large increase in bariatric procedures and the waiving of attempts at clinical therapy in patients with obesity classes II and III (BMI > 35 kg/m^2^) [15] are possibly attributable to the fact that in the past, clinical weight-loss interventions have not been successful enough to avoid bariatric procedures [16, 17]. Indeed, most bariatric surgeries are probably attributable to a lack of suitable clinical alternatives for obesity management [18]. Recent reviews [16–18] have proposed the following possible reasons for the failure of clinical obesity management: (1) Anti-obesity medications are typically administered as monotherapies, even though it is known that none of the available drugs can achieve more than 10% of the weight-loss goal [14, 17]. In contrast, a combination of different medications for obesity may exhibit synergistic effects and provide results that are better than the sum of the weight loss attributable to each drug [17, 18]. Such a synergistic effect has been demonstrated with different drug combinations [19–23], and combination treatment is recommended by the most recent obesity guidelines issued by the American Association of Clinical Endocrinologists (AACE) [24], despite an unwillingness on the part of medical doctors and health insurance companies to apply combined pharmacotherapy, even though this is largely accepted for other disorders (2). In both research and clinical practice [25], pharmacotherapy is not effectively combined with other interventions, such as psychotherapy, surveillance, and intensive diet, even though it is known that the combination of different modalities can achieve tangible and optimized outcomes [25–27]. (3) Pharmacotherapy for obesity is not prescribed over the long term, despite evidence of the safety and benefits of the long-term, on-label use of current anti-obesity medications [27, 28]. (4) There is a lack of weight-loss maintenance strategies, although some studies have proposed successful approaches to prevent weight regain [27, 29]. (5) The complex pathophysiology [27, 30] of obesity and its multiple etiologies that are present in every patient are frequently misunderstood. Such an understanding is critical to provide psychological support [12, 31] allied to intensive lifestyle modification [26], and its lack has arguably led to a decline in diet, physical activity and weight counseling [15]. (6) Anti-obesity medications are greatly underprescribed, as only 2% of patients with obesity (BMI > 30 kg/m^2^) have been prescribed anti-obesity drugs [32].

Regarding the feasibility of clinical interventions to classes II and III of obesity, anti-obesity drugs are able to provide up to 10% of the total weight-loss goal [24, 27], such as the combination of topiramate and phentermine [20], liraglutide [33], combination of bupropion and naltrexone [34], and lorcaserin [35]. Thus, drug treatment cannot induce adequate weight loss in subjects with moderate and severe obesity, if provided as monotherapy and not associated with other treatment modalities [16, 17, 24], but there is enough evidence to show that a multi-disciplinary, intensive and long-term approach and surveillance can lead to results comparable to those of surgical treatments [17, 24–29].

“Lifestyle modifications”, which still rely on general diet counseling and physical activity recommendations, seem to be unhelpful if not intensified and individualized [26]; moreover, they do not provide the minimum weight loss required to achieve risk reduction in most patients [24–27], and do not satisfy patients in the same way as pharmacotherapy and surgery do [36]. Nevertheless, weight-loss strategies have remained mostly unchanged [24, 26, 27], despite evidence of their inefficacy. However, a few new diet strategies, such as intermittent fasting diet and alternate day fasting [37–41], which significantly change the relationship of obese patients with the food-reward system, have shown promising results [37–40].

Considering the aforementioned facts, as well as the unfeasibility to perform bariatric surgery in all recommended patients, due to the high prevalence of morbid obesity (10% women, 8% men and one in every six African American women has a BMI > 40 kg/m^2^ in the USA) [41], we developed a protocol that can potentially overcome the drawbacks that may lead to the failure of clinical therapy for obesity.

Our protocol aimed to improve clinical anti-obesity therapy outcomes, and was therefore based on the following: (1) the identification and improvement of previous approaches, (2) the combination of different strategies that may have synergistic effects, and (3) aggressive clinical intervention. We did not hesitate to combine different modalities and optimize current therapies, as we consider that this approach may be the most effective way to clinically overcome obesity, especially among subjects in whom previous attempts at clinical obesity management have failed.

This protocol may be useful for patients who are considering bariatric surgery but have not yet tried clinical therapy, and may serve as an effective alternative in patients who are unable to undergo bariatric surgery.

The aim of this study is therefore to report the clinical and metabolic effects of our proposed obesity-management protocol over a 2-year period, and to determine whether this more intensive approach to obesity management is feasible and a possible alternative to bariatric surgery in patients with moderate-to-severe obesity.

## Methods

### Proposed interventions

**Fig. 1:**
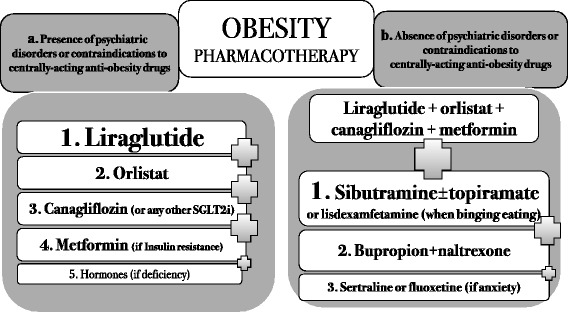
Guideline of pharmacotherapy intereventions against obesity
    There were two possible scenarios for pharmacotherapy: presence or absence of contraindications to centrally acting drugs. For each possibility, a preferred order of drugs was determined, based on the safety and effectiveness profiles of each drug. The combination of all the proposed drugs, whenever feasible, was the first choice of therapy.
    Liraglutide 1.8 mg once a day, orlistat 120 mg B.I.D. before meals, and the sodium glucose transporter-2 (SGLT-2) inhibitor canagliflozin 300 mg once a day were prescribed for all patients, except for those who had contraindications to these drugs or who had not tolerated any of these drugs in previous attempts. Metformin was prescribed at a dose of 2000 mg a day when insulin resistance was found by homeostatic model assessment-insulin resistance (HOMA-IR) > 2.7. Testosterone was prescribed for men with initial testosterone levels <350 ng/dL and no contraindications to testosterone use (i.e., no history of prostate cancer, prostate-specific antigen < 4.0 ng/mL and hematocrit < 50%); 1000 mg testosterone undecanoate (Nebido – Bayer, Germany) was intramuscularly injected every 90 days in eligible subjects. For women with initial testosterone levels below 14 ng/dL (measured on three different occasions to account for inaccuracies in the test) and no history of thrombosis, a dose of 2.0 mg a day was prescribed as a compounded topical cream.
    We screened all patients for psychiatric disorders prior to the prescription of centrally acting medications, as recommended by the AACE guidelines [24]. Subjects who did not present any suspicion of psychiatric disorders and had no contraindications to these classes of drugs were offered the following:
    Sibutramine 15 mg once a day was prescribed for every patient who did not have uncontrolled diabetes, hypertension or high cardiovascular risk, as required by the Brazilian National Health Surveillance Agency (ANVISA), and who had not developed depression with sibutramine use in previous attempts at weight loss.
    Topiramate 50 mg B.I.D. was optionally added to sibutramine, whenever carbohydrate binging was detected. In our clinical practice, we noticed that sibutramine plays a similar role as phentermine when added to topiramate.
    Lisdexamfetamine 50 mg was prescribed when binge eating disorder (BED) was identified, although by the end of the study, we still did not have access to this medication. We screened all subjects for BED, as recommended by the AACE guidelines [24].
    Bupropion 150 mg B.I.D. along with naltrexone 16 mg B.I.D. was prescribed when both carbohydrate binging and excessive alcohol intake were noticed.
    Sertraline 100 mg once a day was offered when anxiety was found to be an underlying cause of obesity.
    The centrally acting medications were also used in combination, except for sibutramine and sertraline (we do not recommend the combination of sibutramine and lisdexamfetamine as well). They were also allied to peripherally acting medications.
  - Phase 2. Slow weaning off of anti-obesity medications
    Specific guidelines or articles about the discontinuation of obesity drugs are almost absent; therefore, all the features of the proposed weaning-off process were based on the rationale of obesity pathophysiology and on the mechanism of action of each drug. The weaning-off process is presented in Figs. 2, 3 and 4.

The proposed interventions include a combination of different strategies, as shown below:

1. Pharmacotherapy
  - Phase 1. Aggressive pharmacotherapy, with a combination of both on- and off-label drugs, according to a guideline that we proposed and is detailed in Fig. 1, allied to regular follow-up with a medical doctor every 2 months.

**Fig. 2:**
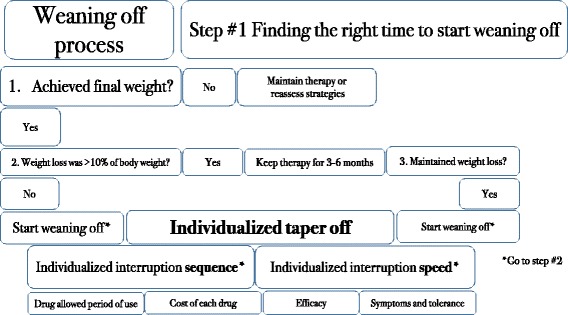
Finding the right moment to start the weaning-off process

**Fig. 3:**
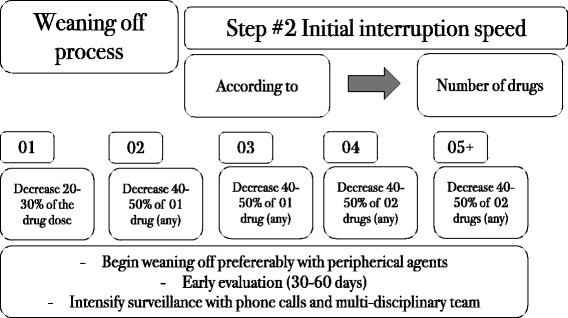
Weaning-off steps

**Fig. 4:**
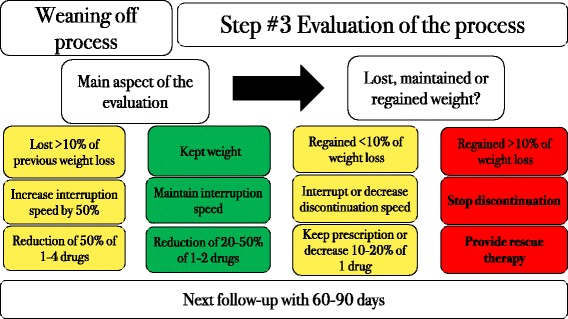
Management of the following steps of the discontinuation process﻿ according to the response to the initial weaning off intervention
  - If a patient regained more than 10% of previously lost weight, he or she was offered a “rescue therapy” (Fig. 5), which consisted of the reintroduction of all medications at the full doses, but for a shorter period. Once weight loss was achieved again, the weaning-off process was restarted, this time at a slower rate.

**Fig. 5:**
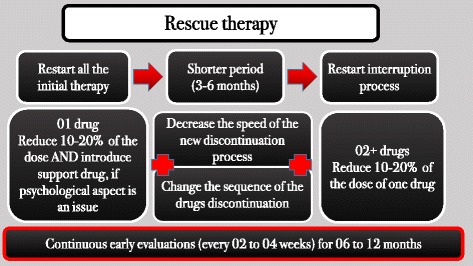
Rescue therapy (in case of weight regain)
2. Non-pharmacological approaches
  Subjects were offered and were required to be followed up for three non-medical modalities:
  - Behavioral therapy. A psychologist provided cognitive behavioral therapy (CBT) that could be done outside the clinic. The adherence criteria were regular follow-up for at least 1 year with 45 min of therapy sessions per week.
  - Exercises. A certified personal trainer, either from the clinical staff or outside, provided monthly follow-up and prescribed exercise regimens that were to be followed at least three times a week. The exercise training consisted of 40 min of moderate-to-intense resistive and cardiovascular exercises (>3 METs, World Health Organization) [42]. Subjects were required to adhere to the training program for at least 10 months in a year (2 months of absence were allowed due to vacations). Subjects had to fulfill the criteria for exercise frequency, regularity and intensity in order to be considered adherents. Additionally, the personal trainers had to provide feedback about the performance and adherence to exercises for each subject, whenever the subjects practiced outside the clinic.
  - Diet prescription. A 500–1500-kcal deficit was aimed for via a hypocaloric diet consisting of 50–55% carbohydrates, 20–25% lipids and 20–30% protein. Additionally, 0.2–0.4 g/kg/day of whey protein was prescribed (whey protein was not taken into account in the calculation of the calorie balance). Subjects were required to attend follow-up appointments with a registered dietician once in every 2 months. A 500-kcal deficit diet was offered in patients with BMI < 40 kg/m^2^, whereas a 1000-kcal deficit diet was prescribed for subjects with a BMI of 40–45 kg/m^2^. A 1500-kcal deficit was prescribed in those with BMI > 45 kg/m^2^. Personalization of diets was performed according to two aspects. First, subjects were actively required to provide individual food preferences as well as most visited restaurants and places to eat. From this, menus and nutritional facts were assessed in order to provide the best options for each place, whenever the chosen meal or place could be included in the diet plan. Second, a general list of foods and ingredients was provided, and subjects had to point out their preferences. With this information, a specific weekly menu was sent to each subject by the dietician. To be considered as adherents, subjects were required to stick to their prescribed diets, in terms of meal sizes and quality, at least 80% of the time and regularly make follow-up visits to a registered dietician (at least six visits yearly).
3. Surveillance
  Intense surveillance was provided as follows:
  - Weekly BW and waist circumference (WC) measurements (every Tuesday) that were sent online to the clinic chart.
  - Monthly body composition analysis with a validated electrical bioimpedance device (InBody770, BioSpace, South Korea) [43–46], air-displacement plethysmography (Bod Pod, CosMed, USA) [47], and 3D body scanning (MyBodee, Styku, USA).
  - Regular phone calls performed every 2 weeks by a clinic staff member, with active inquires of diet, exercise, behavioral therapy and pharmacotherapy adherences.

### Subject selection

This study is a retrospective analysis of an intensive clinical protocol for the management of obesity headed in a private practice clinic that was offered to patients with obesity who searched for an obesity therapy. Included subjects started the protocol from Aug-2013 to Aug-2014 and finished from Aug-2014 to Aug-2015, although they were allowed to voluntarily continue the proposed protocol after the end of the study. The inclusion criteria for this study were as follows: (1) BMI > 40 kg/m^2^ or >35 kg/m^2^ in the presence of comorbidities, such diabetes or hypertension, which would fulfill the TOS/AHA/ACC criteria for bariatric surgery; (2) age between 18 and 70 years; (3) minimum of 1 year of regular follow-up of all the proposed interventions; (4) adherence to at least two of the three non-pharmacological approaches, (5) absence of medications that alter BW, body composition or metabolic parameters (e.g., statins, fibrates, niacin, vitamin E, pioglitazone, sulfonylureas and insulin).

### Clinical outcome measures

The following clinical parameters were evaluated: BW, total weight excess (TWE), BMI, fat weight (FW), muscle weight (MW), WC, visceral fat (VF), and weight regain (WR). BW, VF, MW, and WR were evaluated by InBody 770 [42–45]. FW was analyzed by Bod Pod [46], TWE was estimated by the calculation provided by InBody 770 [44, 46], which considers mineral, water, and muscle masses to determine the ideal weight. This calculation has been validated and can precisely define the amount of excess fat [44–46]. Although body analysis was performed monthly, we used only the initial and final results (at 1 year after the initial results) of body analysis in this study. The body analysis exams were performed monthly in order to improve the level of surveillance. Whenever a lack of FW, VF or BW loss or a gain in any of these measures was observed, nutritional, psychological and pharmacological reassessment was performed in order to identify mistakes in the intervention and modify whatever was identified as improper for the patient.

Subjects were required to perform self-measurements of WC and BW on Tuesdays. Those who did not present WC and BW losses in the first 3 months of the interventions or presented with WC or BW gains at any time, were reassessed. The purpose of this surveillance was to prevent withdrawals and to optimize all aspects of the proposed approach that were not fully personalized to the subjects.

### Metabolic outcome measures

The following markers of lipid and glucose metabolism were assessed: triglycerides (TGs), low-density lipoprotein (LDL) and high-density lipoprotein (HDL) cholesterol, alanine transaminase (ALT), gamma-glutamyl transferase (GGT), glycated hemoglobin (HbA1c), fasting insulin (FI), fasting glucose (FG), uric acid, and C-reactive protein (CRP). HOMA-IR was calculated at the beginning and at the end of the intervention.

The biochemical markers were measured prior to the beginning of the interventions and after 1 year, and were compiled into our study.

The biochemical assays used to measure metabolic markers were as follows: enzymatic assays for TGs and HDL, colorimetric enzymatic assay for uric acid, the modified International Federation of Clinical Chemistry method for ALT and GGT, chemiluminescence for basal insulin, high-performance liquid chromatography for HbA1c, hexokinase assay for glucose, and turbidimetry for CRP. The LDL level was calculated by the Friedewald formula.

The intervention was considered to have been successful, in terms of bariatric surgery avoidance, when 20% of the BW was lost, or the TWE was decreased by at least 50% after 12 months of therapy.

### Statistical analysis

The mean, statististical significance and confidence interval of each variable (clinical and metabolic parameters) were thoroughly analyzed with standardized methods and analysis of variance, by using Microsoft Excel. All data was independently compiled and calculated twice, in distinct documents, and then compared, in order to ensure that there would be no mistakes and therefore provide more certainty and reliability.

### Ethical approval

We do not have an ethics review committee at our institution. We followed the principles outlined in the Declaration of Helsinki. The proposed protocol did not provide new and experimental therapies but combined already standardized modalities, and therefore, did not require approval from an ethics committee.

## Results

### Baseline characteristics

A total of 43 subjects were included in this study (32 women and 11 men). The mean follow-up duration was 17.3 months (+-2.1 months), and the mean BMI at the baseline was 43.08 kg/m^2^ (+-2.66 kg/m^2^). Of the 43 subjects, 13 were taking medications that interfered with the measured metabolic parameters. Thus, the metabolic analysis included only 30 subjects. Twenty (60.5%) subjects had been recommended to undergo bariatric surgery before this clinical intervention. Two subjects withdrew from the therapy, but both were successfully contacted and their results evaluated.

The following medications were prescribed to the study subjects and used regularly throughout the intervention (1 year): liraglutide, 39 (90.7%) subjects; SGLT2 inhibitors, 37 (86.0%) subjects; orlistat, 34 (79.1%) subjects; metformin, 28 (65.1%) subjects; bupropion combined with naltrexone, 25 (58.1%) subjects; sibutramine, 23 (53.5%) subjects; topiramate, 13 (30.2%) subjects; testosterone, 12 (27.9%) subjects; fluoxetine, 11 (25.6%) subjects; and sertraline, 10 (23.3%) subjects. Regarding partial use, one patient used liraglutide for less than 6 months, and one used it for 6–11 months. Five patients used SGLT inhibitors for less than 6 months, and two used metformin for less than 6 months. Four used orlistat for less than 6 months, and three used it for 6–11 months. Two patients used bupropion with naltrexone for less than 6 months, and ten used it for 6–11 months. Sibutramine was used by three patients for less than 6 months, and topiramate was used by four subjects for less than 6 months. One patient used sertraline for less than 6 months. Of the total number of partially used drugs (36, with more than one instance of partial use in some patients), 32 (88.9%) were discontinued due to intolerance to the drugs, while 4 (11.1%) were interrupted with no apparent reason. A summary of this data is exposed in Table 1.

**Table 1:** Pharmacotherapy

MEDICATION	DOSAGE	ON- OR OFF-LABEL?	NUMBER OF SUBJECTS (% OF TOTAL)	PARTIAL USE (NUMBER OF SUBJECTS)
LIRAGLUTIDE	1.8 mg daily	Off-label (during the study period) On-label (2016 Nov)	39 (90.7%)	1 < 6 months 1 6–11 months
SGLT2 INHIBITORS	Canagliflozin 300 mg daily Dapagliflozin 10 mg daily	Off-label	37 (86.0%)	5 < 6 months
ORLISTAT	120 mg B.I.D.	On-label	34 (79.1%)	4 < 6 months 3 6–11 months
METFORMIN	2000 mg daily	Off-label	28 (65.1%)	2 < 6 months
BUPROPION + NALTREXONE	300 mg + 32 mg daily	On-label (USA) Off-label (Brazil)	25 (58.1%)	2 < 6 months 10 6–11 months
SIBUTRAMINE	10-15 mg daily	On-label (Brazil) Prohibited (USA)	23 (53.5%)	3 < 6 months
TOPIRAMATE	100 mg daily	On-label (USA) (when combined with phentermine) Off-label (Brazil)	13 (30.2%)	4 < 6 months
TESTOSTERONE	1000 mg I.M. every 3 months	On-label (when hypogonadism is present)	12 (27.9%)	-
FLUOXETINE	20-60 mg daily	Off-label	11 (25.6%)	1 < 6 months
SERTRALINE	50-200 mg daily	Off-label	10 (23.3%)	1 < 6 months

A total of 41 (95.3%) subjects adhered to the diet plans according to the proposed criteria, whereas 33 (76.7%) subjects underwent physical therapy according to the proposed conditions, and 16 (37.2%) underwent regular psychotherapy as stated in the protocol. Four subjects (9.30%) underwent all the non-pharmacological approaches regularly, and 39 (90.70%) followed two of the three non-medical modalities. Although not specified in the study, subgroup analysis of adherence to the various therapies, i.e., physical therapy and psychotherapy, physical therapy and diet plan, psychotherapy and diet plan, and to all three approaches did not show any statistical differences in both clinical and metabolic outcomes between these groups.

### Clinical outcomes

BW, BMI, FW, TWE, WC, and VF were all significantly reduced after 1 year of the intervention as compared with the baseline (Table 2). MW was decreased by 2.9 kg on average, and accounted for only 9.2% of the total weight loss. Thus, 82.1% of BW loss was from the loss of fat mass. All 43 subjects (100.0%) achieved a minimum of 5% BW loss. Thirty-eight (88.4%) subjects lost >10% of their BW, while 32 (74.4%) lost >20% of their BW (among which, only 5 subjects lost <50% of their TWE). In total, 35 (81.4%) subjects lost more than 50% of their TWE. However, eight of these did not lose more than 20% of their BW. Twenty (46.5%) subjects achieved their desired WC (<94 cm).

**Table 2:** Clinical responses to intervention

	Baseline	After intervention	Change
Body weight (kg)	121.6	90.3	−31.3 (−25.7%; *p* < 0.001)
BMI (kg/m^2^)	43.08	31.99	−11.09 (−25.7%; *p* < 0.001)
Fat weight (kg)	55.4	29.7	−25.7 (−46.4%; *p* < 0.001)
Total weight excess (kg)	45.2	18.1	−27.1 (−60.0%; *p* < 0.001)
Waist circumference (cm)	131.2	99.4	−23.1 (−17.6%; *p* < 0.001)
Visceral fat (cm^2^)	263.8	101.0	−162.8 (−57.9%; *p* < 0.001)

**Fig. 6:**
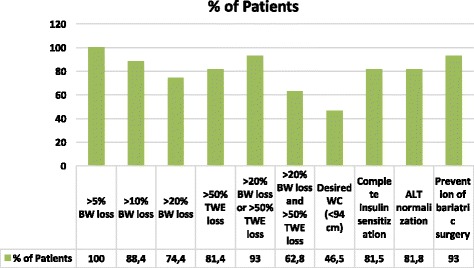
Goals achieved by patients

All 43 patients were initially candidates for bariatric surgery. Of these, three (7.0%) subjects did not achieve sufficient BW loss to avoid this procedure, and were therefore referred to a bariatric surgeon. The remaining 40 (93.0%) subjects continued to undergo clinical follow-up after achieving 20% BW loss or a 50% decrease in TWE, or both. In 31 (72.1%) subjects, the BMI was decreased by at least two classes of the BMI obesity classification. The overall goals achieved, in terms of both clinical and metabolic outcomes, have been shown in Fig. 6.

### Metabolic outcomes

The TG, LDL cholesterol, ALT, GGT, BI, FG, HOMA-IR, HbA1c, uric acid, and CRP values all significantly decreased after 1 year of the intervention as compared with the baseline (Table 3). The HDL level did not change significantly. Among the 30 evaluated subjects, 22 had abnormal ALT levels at the baseline (normal range, 7.0–43.0 U/L). After the intervention, only four of these subjects continued to have abnormal ALT levels, yielding a remission rate of 81.8% (*p* < 0.001). The HOMA-IR value was elevated in 27 patients and normalized in 22 patients, yielding a recovery rate of 81.5% (*p* < 0.001) for insulin sensitivity.

**Table 3:** Metabolic findings after clinical intervention

	Baseline	After intervention	Change
TG (mg/dL)	177.4	81.0	−96.4 (−54.3%; *p* < 001)
LDL (mg/dL)	118.1	98.8	−19.3 (−26.3%; *p* < 0.05)
ALT (U/L)	52.9	27.8	−25.1 (−47.4%; *p* < 0.001)
GGT (mg/dL)	47.8	15.2	−32.6 (−68.2%; *p* < 0.001)
Basal insulin (μIU/mL)	23.1	7.9	−15.2 (−65.8%; *p* < 0.001)
Fasting glucose (mg/dL)	91.4	77.5	−13.9 (−15.2%; *p* < 0.001)
HOMA-IR	5.21	1.51	−3.7 (−70.8%; *p* < 0.001)
Uric acid (mg/dL)	7.1	5.7	−1.4 (−19.7%; *p* < 0.001)
CRP (mg/L)	0.73	0.39	−0.34 (−46.6%; *p* < 0.001)
HbA1c (%)	5.87	5.15	−0.72 (−13.6%; *p* < 0.001)

## Discussion

### Pharmacotherapy

Historically, anti-obesity pharmacotherapy has been improperly managed due to (a) the too short-term drug prescriptions [16, 28, 29, 48], (b) lack of weight maintenance follow-up [16, 27, 29, 49, 50], (c) lack of pharmacological combinations [16–18, 30], and (d) undertreatment [16, 51], since only 2% of patients with obesity are prescribed pharmacological treatments [51]. Obesity involves multiple central and peripheral mechanisms to prevent weight loss and induce weight regain after a period of weight loss, such as decreased resting metabolic rate and enhanced food-reward circuit sensitivity [16, 27, 29, 49]. Thus, a combination of multiple drugs, whenever there are no harmful interactions, is more adequate and effective than monotherapy, as demonstrated in several studies [16–25]. Likewise, long-term therapy tends to be more successful, particularly, for weight-regain prevention [48], given the fact that metabolic adaptations after weight loss tend to persist for several years afterwards [27, 29, 52], which makes weight loss maintenance challenging [16, 27–29, 49, 50]. Indeed, the AACE obesity guidelines recommend the avoidance of short-term therapy (less than 6 months) due to the proven safety profile and superiority of the long-term use of all the available anti-obesity drugs. The guidelines also recommend a combination of drugs [24], as “appetite regulation involves multiple pathways, and targeting more than one pathway concurrently may have additive or synergistic effects” [24].

The proposed pharmacotherapy protocol in the present study, therefore, relied on the long-term prescription of a combination of many medications. The prescribed non-centrally acting drugs (medications that do not alter neurotransmitter production or signaling) were as follows:

1. Liraglutide, a GLP-1 analogue, which has been approved for long-term obesity treatment [33, 48], and has wide peripheral and central actions, including a central action on newly discovered GLP-1 pathways [53–55];
2. Orlistat, a lipase inhibitor, has been extensively proven to be safe and effective, and has exhibited other benefits besides weight loss, such as effects on non-alcoholic steatohepatitis (NASH) [55] and glycemic control [56–58];
3. SGLT2 inhibitors, which are anti-diabetic drugs that promote weight loss due to glycosuria and calorie loss through urine; they were used here as off-label therapy for obesity. Additionally, the SGLT2 inhibitor empagliflozin has been shown to reduce cardiovascular risks [59], an effect that is expected to be common to this entire class of drugs [60]. SGLT2 inhibitors are also effective for weight loss in non-diabetic patients [61], and have probable synergistic effects with GLP-1 analogues on both glycemic control and weight loss [61]. SGLT2 inhibitors induce gluconeogenesis and increase glucagon and GLP-1 levels, which may contribute to fat loss [62]. In fact, it is been found that SGLT2 inhibitors can be effective as monotherapy for non-diabetic subjects and can enhance weight loss as an add-on therapy to GLP-1 analogues [63];
4. Metformin, a muscle and liver insulin sensitizer, can promote weight loss, especially when insulin resistance is found [64, 65]; and
5. Testosterone was prescribed whenever its deficiency was detected and there were no contraindications, as it has been shown to promote fat loss when hypogonadism and obesity are present together, and improves several metabolic parameters, particularly, in men [66, 67].

Among centrally acting drugs, the proposed medications used in this study were as follows:

Sibutramine, a noradrenaline and serotonin reuptake inhibitor, has been prohibited in some countries, but its use is still allowed, under strict control, in Brazil. The prohibition of sibutramine in some countries was due to the SCOUT study [68], which showed an 11% increase in cardiovascular events with sibutramine use. However, several issues were found in regards to the design of this trial: (a) subjects who were at a high risk of cardiovascular disease were included, which is questionable since a high cardiovascular disease risk is a relative contraindication to sibutramine use; (b) the medication was maintained in patients who did not exhibit weight loss, despite the standardized recommendation of interruption of sibutramine in case of non-responsiveness; (c) subgroup analysis, although not the primary objective, showed that among subjects who were at a high risk of cardiovascular disease, weight loss was associated with a decrease in this risk, whereas among subjects who were not at a high risk of cardiovascular disease, no correlation was detected between weight loss and cardiovascular disease risk. Indeed, further studies involving post-hoc analysis [69] and studies with large populations [70] showed that the wide prohibition of sibutramine marketing might have been inappropriate for patients without cardiovascular disease;

1. Topiramate, originally developed as an anti-epileptic agent, had its use extended to obesity management, when combined with phentermine [20]. This is a safe drug combination [71] that reduces carbohydrate craving and calorie intake [72]. Topiramate also reduces insulin [20] and leptin [73] resistances, and has been shown to exert direct lipolytic effects [74]. Topiramate as an add-on therapy to sibutramine was a plausible alternative in our practice, as phentermine is still not approved in Brazil.
2. Bupropion with naltrexone, which is a combination of a noradrenaline and dopamine reuptake inhibitor and an opioid receptor antagonist with synergistic effects, has been approved as an anti-obesity therapy. It has been shown to induce even greater weight loss than previously predicted (−9.2 kg versus −6.6 kg in previous studies) [75]. Additionally, it reduces CRP and WC, and increases HDLc [75].

Lorcaserin, a 5-HT2c receptor agonist, was not included in our protocol, since this drug is not officially approved in Brazil, despite its proven safety and efficacy in different studies [35, 76, 77] and a possible effect on BED [78–81]. We recently included lisdexamfetamine in our protocol, even though the current study does not have any subject in whom the drug was used. Lisdexamfetamine has been shown to be effective against BED [82, 83], which has been confirmed by a systematic review of randomized controlled trials [84]. Several newly discovered brain pathways that induce obesity [85–89] reinforce the importance of centrally acting drugs in obesity, preferably targeting more than one mechanism.

### Diet

A personalized diet plan was employed for each subject, according to their food preferences and social environment. Studies have shown that non-adherence to diets occurs when individual aspects are not taken into account [26, 27, 31]. The addition of whey protein was based on the findings of several studies that showed that this source of protein can prevent fat gain with high-fat diets, enhance fat loss with hypocaloric diets and considerably improve metabolic parameters [90–93].

We did not require 100% diet adherence to classify the subjects as adherents to the proposed diet plans. It has been observed that intermittent diets, such as alternate day fasting and intermittent fasting diet, allow periods of free meals and have no negative impact on weight loss [37–40] when the rest of the diet plan is strictly followed. These diets also help to decrease long-term BED [40]. When 100% diet adherence is not required, social events and travels become feasible, as both situations induce diet escapes and further loss of adherence to food recommendations.

BED is an expected disorder among patients with obesity, particularly, in patients with BMI > 35 kg/m^2^, more than 50% of whom are affected by compulsive eating disorders [27]. The active search for BED in these patients is supported by AACE obesity guidelines [24].

### Exercise

Although exercises may not be the best approach to promote weight loss in obesity, they do improve fat loss, help avoid muscle loss and prevent weight regain by increasing energy expenditure and decreasing BED symptoms [27]. Intensive surveillance of adherence to physical activity was critical for the success of the proposed interventions.

### Behavioral therapy

The role of behavioral therapy in obesity management is well established [29–31], especially for weight maintenance, when motivation decreases, as behavior plays a critical role in the pathophysiology of obesity. Conversely, psychological modalities of obesity management are being decreasingly used [15], possibly due to the lack of professional education on obesity [31]. Among the various types of psychotherapies, the behavioral cognitive approach is the most studied strategy for weight loss and has therefore been standardized for obesity management [30, 31].

The lack of adherence to regular follow-up with psychotherapists was possibly the result of a misbelief that the behavioral approach is not necessary nor effective, despite strong evidence of its efficacy [31]. We did not require a psychotherapist who specialized in obesity, since it is not feasible to conduct large-scale obesity therapy with obesity specialists due to the high prevalence of this disorder. The only requirement was that subjects be followed up with CBT approaches, based on the evidence of this strategy of obesity management.

### Surveillance

This study proposed three types of surveillance: the assisted body analysis monthly surveillance, the regular phone calls and the weekly self-surveillance. They were employed independently in order to provide more intense oversight. The importance of surveillance on the efficacy of weight loss and maintenance strategies has been broadly studied [94–98] and is a key aspect in the success of both weight loss and maintenance.

Weekly self-surveillance was recommended to be performed on Tuesdays, and not on Mondays, since weekends are the periods when patients are more likely to increase carbohydrate and sodium intakes, with consequent accumulation of glycogen (in the liver and muscles) and retention of water, respectively. The excessive carbohydrate and sodium intakes can add up to 3–4 kg of extra weight, but this is quickly lost on the first day of adequate diet, usually on Mondays [99]. Phone calls have also been reported play a significant role in ensuring long-term adherence to obesity therapy [100], which was confirmed by us.

#### Assessments of responses to obesity-management therapies

The assessment of responses to obesity therapy should go beyond mere BW measurements, and include evaluations of metabolic parameters, WC, and FW, which are accurate predictors of cardiovascular and metabolic risks [27, 29]. BMI may not be an accurate parameter and can mislead interventions [101–103]. One of the best ways to evaluate the response to obesity-management therapies is to analyze markers of glucose and lipid metabolism and inflammatory markers, as these will more directly predict risk reductions. Good clinical methods, such as body composition analysis and WC measurements, also improve the accuracy of response assessments. For example, the prevention of lean mass loss is an important goal of obesity management, and this parameter is underestimated when BW is the only outcome analyzed. Lean mass can be determined using body composition analysis and estimated using WC. Approaches that preserve muscle mass, such as resistive exercises, tend to be underappreciated when weighing scales are the only tool employed. Furthermore, the effectiveness of strategies that induce intensive loss, such as bariatric procedures, is overestimated by weighing scale measurements, owing to intense muscle loss.

To ensure the quality of the methodology and the accuracy of the results, it was important to exclude from the metabolic analysis subjects who received medications that improved metabolic markers. These improvements were not related to the proposed intervention protocol, and could falsely improve the final results. However, patients receiving *on-* and *off-label* drugs for obesity that also improve metabolic markers were included in the metabolic analysis, as they were prescribed these drugs regardless of their baseline metabolic levels.

#### Metabolic and clinical outcomes

After the intervention, impressive improvements were seen in markers of liver function, lipid metabolism, glucose metabolism, and inflammation, and were better than the improvements previously described in the literature [16–22, 33–35]. It is important to note that none of the medications used in this study is indicated for the correction of liver dysfunction. Therefore, the improvements observed were secondary to FW loss and the beneficial side effects of some of the drugs. The significant clinical and metabolic improvements observed in this study will probably reduce overall risks [27], an effect which is enhanced by the inherent protective cardiovascular effects of some of the medications used in this protocol, such as liraglutide [32], SGLT inhibitors [59], metformin [65] and orlistat [19, 29].

Although anti-hyperglycemic medications were prescribed, HbA1c dropped more than expected. The initial HbA1c was less than 6.0%, and it is known that the lower the HbA1c level, the harder it is to decrease. The reduction in inflammatory markers, such as CRP and uric acid, was also attributable to general metabolic changes, as no specific drugs were prescribed for these markers. The improvements observed in this study were greater than the sum of the previously described improvements attributable to each of the prescribed drugs [20, 33–35]. Thus, synergistic effects of the different drugs used in this study may explain these surprising positive findings.

Normalization of liver transaminases was seen in most patients, suggesting that clinical obesity management may be attempted prior to specific etiological investigations and therapies for NASH. This approach may also prevent unnecessary invasive procedures (such as liver biopsy). Further studies investigating the quantitative imaging and classification of NASH before and after anti-obesity therapy, together with biochemical liver-function analysis are recommended.

The highly selective loss of fat mass was possibly due to the intensive body constitution surveillance and consequent adjustments in terms of diet and exercise plans (and also due to testosterone therapy in patients with hypogonadism), which probably led to more significant changes in metabolic parameters and WC.

Finally, this study showed that patients do not necessarily need to follow up with all three non-pharmacological interventions (CBT, physical therapy and diet plan) and do not have to specifically follow certain therapies, since there were no differences in results among the various therapy groups, regardless of whether two or three interventions were regularly followed, or between different combinations. Despite being the ideal approach, follow-up with all three modalities can be tiring and time-consuming, and patients are therefore less likely to adhere all of these treatments in the long run.

### Changes in anti-obesity strategies

Instead of introducing one intervention at a time and evaluating the response to each intervention, we optimized all the key aspects of the proposed protocol at once, given that: (1) obesity is a complex, hard-to-manage disease, (2) its prevalence and severity are quickly increasing, and (3) many issues have been identified regarding the proposed current therapies. Furthermore, aggressive approaches induce greater weight loss, which can positively predict long-term weight loss and maintenance [104].

In our opinion, obesity is a too severe a disorder for step-by-step approaches. It has several long-term consequences, including a more than 500% increase in cardiovascular disease, 260% increase in overall mortality after 18 years of obesity [105], and increased public health costs in more than 50% of subjects [106]. Owing to these reasons, we considered an aggressive approach to obesity management to be more suitable. Once the proposed combination of interventions is demonstrated to be effective, then we suggest that the number, intensity and agressiveness of the interventions be slowly decreased until an optimal protocol that remains chronically effective is found.

Besides developing an aggressive therapeutic protocol combining on and off-label medications, we were also concerned with determining the optimal time period for which medications should be taken as well as designing a suitable discontinuation plan. We could not find any information in the literature about when and how obesity drugs should be weaned off. We therefore developed a protocol based on the mechanisms of action and safety profiles of the proposed drugs.

**Fig. 7:**
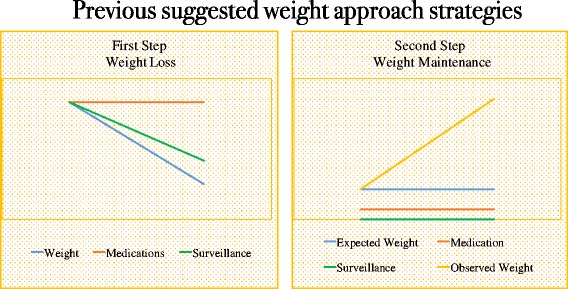
Previous paradigm on steps of obesity management

**Fig. 8:**
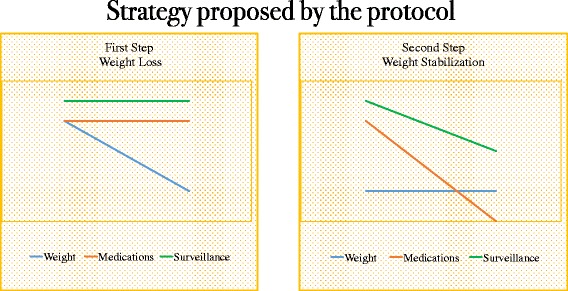
First and second steps of obesity management proposed by the present protocol﻿: 1. Weight loss; and 2. Weight stabilization

**Fig. 9:**
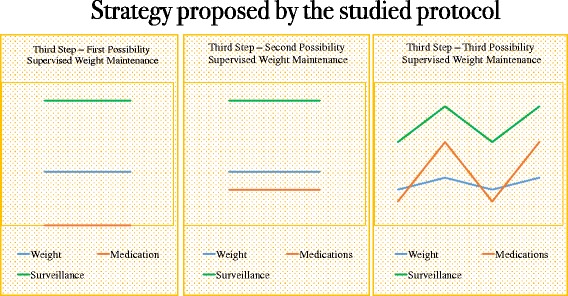
Weight maintenance strategies to prevent weight regain

Although previous studies [10, 15, 24, 29] have recommended a two-step approach (weight loss and weight maintenance) to obesity management (Fig. 5), there is a crucial period between these two steps that cannot be part of either of these periods. Herein, we named this in-between period as weight stabilization. This step starts right after the achievement of the final body weight, and the length of the weight-stabilization period will depend on the amount of weight loss and duration of previous obesity. The longer obesity was present, the longer it takes to stabilize the new weight. It is important to point out that duration of this period is estimated and therefore not precise, as there is no accurate predictor of how long a weight-stabilization period would be necessary in each case. Our strategy has been illustrated in Figs. 6, 7, 8 and 9 to provide an easier understanding of the proposed therapy. The intervention was continued after the end of the first year (the period of this study) by 42 of the 43 subjects (one subject moved out), once they understood the importance of long-term therapy, and with whom we are currently applying the proposed steps for weight loss, stabilization and supervised maintenance.

Rescue therapy was performed in two patients during the study. One of them had lost 26.7 kg but regained 6.9 kg during the weaning-off process, after his mother’s death; all the medications were re-prescribed in their full doses. The other subject had an exacerbation of BED, and consequently regained 9.3 kg of the 22.0 kg of previously lost weight. We reintroduced all the medications and added lisdexamfetamine at a daily dose of 50 mg. Both patients were able to fully lose the regained weight in a period of less than 3 months, which reinforces the importance of a prompt approach once weight regain is detected.

We consider it important to maintain as many aspects of real life as possible during the proposed intervention, for example by including programmed interruptions in physical activity, allowing vacations and not having an obligation for 100% adherence to diet. We consider that there is a theoretically higher chance of long-term success when individuals with obesity do not stand apart from their realities while undergoing obesity therapies. All 43 subjects had tried to lose weight medically at least twice, with medications, diet and exercises. However, the amounts of the previous weight losses were less intense and the duration of the weight losses were short, usually less than 3 months (in 38 of 43 subjects).

The cost of the proposed therapy for a 2-year period is five times less than the expected increase in costs due to obesity complications [107–109]. Once effective weight loss occurs, the protocol would be able to decrease costs by more than US$600 billion over the next 20 years [110]. Even though current approaches are still unable to provide long-term reduction in health costs due to weight regain, which is observed with most approaches and enforces the rationale for the current lack of health insurance coverage for obesity treatments [110].

Herein, we proposed a different way to express weight loss: controlled obesity, not ex-obesity, similar to diabetic patients in whom glucose is normalized or to hypertensive patients in whom blood pressure is controlled. The practical difference in terms of naming the weight-maintenance period as “controlled obesity” is that it helps support the notion of long-term therapy, regardless of the types of intervention, as obesity is seen as a current disease even in weight-controlled subjects.

### Weight maintenance

Obesity approaches are often successful in inducing weight loss, but not in preventing weight regain [29, 49], due to several reasons: (1) no protocols or guidelines are available on drug weaning-off strategies; (2) a standardized optimal period of drug use is lacking; pharmacological strategies vary according to the clinical judgment of the treating physician, and are not based on any previous protocol or studies; (3) there is a loss of motivation after the achievement of the final weight, when the positive reward of losing fat fades away; and (4) there is a lack of long-term studies with currently approved interventions.

#### The need of effective clinical approaches to obesity management

The high prevalence of moderate and severe obesity [41], particularly among lower income and scholarship subjects [41] turns unfeasible to provide proper bariatric surgery and follow-up to the whole population that has formal indication for this procedure. Given this fact, an effective clinical approach could be an alternative to help millions of subjects with obesity.

Therefore, the objective of this study was to develop an effective, holistic, clinical protocol for obesity management that can be implemented before bariatric surgery. We developed this protocol by correcting the historical mistakes in obesity interventions generally observed in clinical studies, such as the dissociation between pharmacological and non-pharmacological strategies, and the lack of combination drug therapies. We speculated that these reasons could explain why clinical therapy for obesity often fails.

#### Clinical management vs. bariatric surgery

To our knowledge, this is the first study to analyze an intensive clinical approach to obesity management, incorporating combined pharmacotherapy and non-pharmacological modalities as well as the systematic evaluation of clinical and metabolic parameters. Indeed, the commitment of the whole team to the protocol, the quality of the diet plans, the monitoring of the adherence to exercise regimens and psychotherapy combined with continuous body surveillance allowed this clinical protocol to serve as an alternative to bariatric surgery.

Although we did not perform a head-to-head comparison, the outcomes of this protocol are comparable to those of bariatric surgeries, especially to sleeve gastrectomy, and indicate that clinical strategies can be effective in obesity management, especially when optimal and synergistic strategies are selected. Our search for an effective strategy to avoid bariatric surgery was an attempt to cease and possibly reverse the recent trend of the trivialization of bariatric surgery. This trend is worrisome because post-bariatric surgery patients must take lifelong precautions that are not required from non-surgical ex-obese subjects [13, 14, 30]. In our medical practice, we found that many post-bariatric surgery patients were not always aware of the possible complications of the surgery and indeed, of the patients responsibilities, which is corroborated by previous studies [13, 14].

#### Limitations of this study

We are aware that the proposed protocol is not easily reproducible, not due to biological issues, but due to social and financial conditions of obesity therapy centers and affected subjects. The drugs used are expensive and are not usually covered by health insurance in the USA and in Brazil. Furthermore, pharmaceutical industries produce each of the proposed medications in separate packages, which hampers the conducting of studies with potential drug combinations. In fact, several issues in fighting obesity with pharmacotherapy were highlighted by a study [16], such as not treating obesity as a chronic disease, lack of availability of other clinical strategies for weight loss, culturally unacceptable adverse effects of anti-obesity agents, low sales performance of obesity medications (which weakens the research for new molecules), and lack of drugs covered by health insurance.

Besides the difficulty in reproducing this study, other important limitations of our paper are the lack of a control group, possibility of enhanced effects due to the placebo effect, small number of subjects, and the retrospective nature of the analysis, although none of the patients were lost to follow-up. Despite these limitations, the impressive results observed in the subjects are hardly questionable, and support the hypothesis that anti-obesity interventions should be aggressive, although this must be further evaluated in controlled trials in future.

#### Final discussion

The results of this protocol highlight the feasibility of clinical anti-obesity therapies in patients with moderate-to-severe obesity as well as the need for a multidisciplinary and aggressive clinical approach to the patient with obesity prior to bariatric surgery, as this has been shown to be more effective than isolated treatments. Anti-obesity therapy, regardless of the type of practice, should be offered as a combination of different strategies, and pharmacotherapy must be a part of this therapy in order to provide effecrtive management. Our anti-obesity approach is not easy to be implemented, as it requires several professionals, and strict and continuous contact with patients; however, as the results show, our approach may be a good alternative for patients prior to bariatric procedures. Secondary findings are also important to note, such as remission of altered liver profile and improvements in several metabolic disorders, which confirm the efficacy of the proposed approach.

## Conclusion

An intensive and aggressive clinical approach to obesity management can be an effective alternative to bariatric surgery, although further studies are required to confirm our findings.

## Abbreviations

AACE: American Association of Clinical Endocrinologists
ACC: American College of Cardiology
AHA: American Heart Association
ALT: Alanine transaminase
ANVISA: Brazilian National Health Surveillance Agency
BED: Binge eating disorder
BMI: Body mass index
BW: Body weight
CBT: Cognitive behavioral therapy
CRP: C-reactive protein
FG: Fasting glucose
FI: Fasting insulin
FW: Fat weight
GGT: Gamma-glutamyl transferase
HbA1c: Glycated hemoglobin
HDL: High-density lipoprotein
HOMA-IR: Homeostatic model assessment-insulin resistance
LDL: Low-density lipoprotein
MW: Muscle weight
NASH: Non-alcoholic steatohepatitis
SGLT-2: Sodium glucose transporter-2
TG: Triglycerides
TOS: The obesity society
TWE: Total weight excess
VF: Visceral fat
WC: Waist circumference
WR: Weight regain
